# Fractional analysis of unsteady squeezing flow of Casson fluid via homotopy perturbation method

**DOI:** 10.1038/s41598-022-23239-0

**Published:** 2022-11-01

**Authors:** Mubashir Qayyum, Efaza Ahmad, Sidra Afzal, Tanveer Sajid, Wasim Jamshed, Awad Musa, El Sayed M. Tag El Din, Amjad Iqbal

**Affiliations:** 1 Department of Sciences and Humanities, National University of Computer and Emerging Sciences, Lahore, Pakistan; 2 Department of Mathematics, Capital University of Science and Technology (CUST), Islamabad, 44000 Pakistan; 3 Department of Physics, College of Science and Humanities in Al-Aflaj, Prince Sattam Bin Abdulaziz University, Al-Aflaj, 11912 Saudi Arabia; 4 Department of Physics, College of Science, Sudan University of Science and Technology, Khartoum, Sudan; 5 Electrical Engineering, Faculty of Engineering and Technology, Future University in Egypt, New Cairo, 11835 Egypt; 6 Department of Materials Technologies, Faculty of Materials Engineering, Silesian University of Technology, 44-100 Gliwice, Poland; 7 CEMMPRE—Centre for Mechanical Engineering Materials and Processes, Department of Mechanical Engineering, University of Coimbra, Rua Luı’s Reis Santos, 3030-788 Coimbra, Portugal

**Keywords:** Mathematics and computing, Physics

## Abstract

The objective of this article is to model and analyze unsteady squeezing flow of fractional MHD Casson fluid through a porous channel. Casson fluid model is significant in understanding the properties of non-Newtonian fluids such as blood flows, printing inks, sauces and toothpaste etc. This study provides important results as unsteady flow of Casson fluid in fractional sense with aforementioned effects has not been captured in existing literature. After applying similarity transformations along with fractional calculus a highly non-linear fractional-order differential equation is obtained. Modeled equation is then solved along with no-slip boundary conditions through a hybrid of Laplace transform with homotopy perturbation algorithm. For validity purposes, solution and errors at various values in fractional domain are compared with existing results. LHPM results are better in terms of accuracy than other available results in literature. Effects of fractional parameter on the velocity profile, skin friction and behaviors of involved fluid parameters is the focal point of this study. Comprehensive, quantitative and graphical analysis is performed for investigating the effects of pertinent fluid parameters on the velocity profile and skin friction. Analysis revealed that fractional parameter depicts similar effect in case of positive and negative squeeze number. Also, skin friction decreases with an increasing fractional parameter. Moreover, in fractional environment Casson parameter has shown similar effect on the velocity profile in case of positive and negative squeeze number.

## Introduction

Fractional calculus deals with non-integer order derivatives and integrals. In 1695, concept of fractional differentiation emerged when L’Hospital sent a letter to Leibniz asking about 1/2th order derivative. Many well-known mathematicians such as Riemann^1^, Liouville^2^, Abel^3^, Euler^4^, Laurent^5^, Laplace^6^, and Hardy^7,8^ played a vital role in the foundation of fractional calculus. This area of mathematics acquired attention in late twenties due to its vast industrial applications. In 1974, first international conference on fractional calculus was held and in the same year first textbook dedicated to this field was published^9^. Fractional calculus has variety of important roles in various fields of science and engineering such as diffusion procedures, geochemistry, viscoelasticity, and bio-engineering etc. It is also an efficient way to model complex problems such as fluid flow, electromagnetic theory, decentralized wireless networks, biology, physics, micro-grids etc. In literature, many fractional order derivatives were proposed to solve real world problems of different fields^10–13^. In recent years, mathematical models with fractional order derivatives gathered a lot of attention because they provide a better fit to the real data as compared to integer-order models^14–18^. This is observed in fractional order modeling of biological systems which provides a much deeper understanding of the complex behavioral patterns of many infectious diseases like avian influenza^19^, dengue fever^20^, malaria^21^, TB^22^ and Hepatitis B^23^ etc.

In fluid dynamics, squeezing flow between parallel plates is considered to be an important due to its involvement in many applications models^24–28^. This type of flow is similar to the principal of moving pistons in heavy mechanical machinery. Applications of these flows can be seen in automobile engines, hydraulic machinery, and food industry etc. models^29–32^. Another important example is patterns in fluid flow. These patterns are usually classified into turbulent, transitional and laminar flows. In research community, the effects of these behaviors on non-Newtonian fluids have proved to be a significant challenge. The study of magneto-hydrodynamic (MHD) effect, which is related to fluid behavior under applied magnetic field, has been a crucial topic of interest in fluid flow. Some examples of fluids that follows MHD effect are plasmas, electrolytes, salt water, etc. Due to its diverse behavior in different fields of science like geophysics, cooling systems, MHD generators, etc., many researchers take interest in it models^33–35^. In current study, a non-Newtonian Casson fluid model^36,37^ is taken, which is able to capture complex rheological properties of different concentrated fluids like syrups, toothpaste, juices, printing inks, honey etc. Casson fluid is interpreted as a shear thinning liquid which is usually assumed to have infinite viscosity at zero rate of shear, and no flow occurs below its yield stress^38–40^.

Due to presence of fractional derivatives and non-linearity of the models, exact solutions are rare. Hence, numerical or semi-numerical approach is required for prediction and analysis instead of analytical approaches^41^. Usually for boundary value problems (BVPs), perturbation techniques are used, but due to presupposition of some small or large parameters, these techniques are insufficient for complex phenomena. Considering this fact, Prof. He merged perturbation tools with homotopy, and named it homotopy perturbation method (HPM)^42^. This scheme is then applied by many researchers in different fields of science and engineering^43–45^. In order to reduce errors and improve the efficiency of classical HPM, different modifications are also proposed by researchers. Few of the recent alterations of HPM can be seen in^46–48^. Among these modifications, one is Laplace homotopy perturbation method (LHPM) which is obtained by combining HPM with Laplace transform. Laplace transform is much important in finding the solutions of differential equations. Better accuracy is achieved by combining Laplace transform with HPM. Moreover, it provides convergent series solution without discretization and hence can be easily applicable to wide variety of problems LHPM was introduced by Johnston^49^ for finding the solutions of fractional Burger equations. Li and Nadeem used He-Laplace method in shallow water waves^50^. In this paper, authors extend this method to solve and analyze unsteady squeezing flow of fractional Casson fluid model with MHD and porosity effects. The objective of this paper is to investigate the effect of fractional parameter on fluid velocity, skin friction and different fluid parameters ($\beta ,{M}_{g},{M}_{p},{S}_{q}$). Moreover, effect of fluid parameters ($\beta ,{M}_{g},{M}_{p},{S}_{q}$) on the velocity profile in fractional and integral environment is also investigated in this study.

In rest of the paper, “Mathematical formulation” and “Laplace homotopy perturbation method for fractional differential equations” sections consists of mathematical formulation and basic idea of LHPM for fractional order boundary value problem respectively, “Application of LHPM to fractional squeezing of Casson fluid” section consists of application of LHPM to fractional squeezing of Casson fluid, “Results and discussion” section presents results and discussion while conclusion is given in “Conclusion” section.

### Basic definitions

#### **Definition 1**

The Laplace transform $\mathfrak{L}[\mathcal{U}(\zeta )]$ of Riemann–Liouville fractional integral ${\mathfrak{I}}^{\alpha }$^51^ is given as follows^49^:

1 $$\mathfrak{L}[{\mathfrak{I}}^{\alpha }\mathcal{U}(\zeta )]={s}^{-\alpha }\mathfrak{L}[\mathcal{U}(\zeta )].$$

#### **Definition 2**

The Laplace transform $\mathfrak{L}[\mathcal{U}(\zeta )]$ of Caputo’s fractional derivative ${\mathfrak{D}}^{\alpha }$^51^ is given as follows:

2 $$\mathfrak{L}[{\mathfrak{D}}^{\alpha }\mathcal{U}(\zeta )]={s}^{\alpha }\mathfrak{L}[\mathcal{U}(\zeta )]-\sum_{n=0}^{k-1}{s}^{\alpha -n-1}{\mathcal{U}}^{(k)}(b), k-1<\alpha \le k.$$

## Mathematical formulation

Consider an in-compressible flow of Casson fluid between two plates that are initially at the distance $\mathcalligra{l}$. At time $t$, the distance between these two plates is $\widetilde{z}=\pm \mathcalligra{l}\,\,(1-\gamma t{)}^{1/2}=\pm \widetilde{h}(t)$. Suppose $\gamma$ is the squeezing or receding motion of the plates. $\gamma >0$ corresponds to a squeezing while $\gamma <0$ represents the receding motion of the plates. This phenomena is of much importance in literature as it is equivalent to the blood flow through arteries, heart and squeeze motion of lungs etc.^52–54^. $t=\frac{a}{\gamma }$ is the time when both plates touch each other. Considering these conditions, the non-Newtonian Casson model^55^ is

3 $${\tau }_{ij}=\left\{\begin{array}{l}2[{p}_{y}/2\pi +{\mu }_{A}]{e}_{ij}, \;\; \pi >{\pi }_{c}\\ 2[{p}_{y}/2{\pi }_{c}+{\mu }_{A}]{e}_{ij}, \;\;{\pi }_{c}>\pi .\end{array}\right.$$

where ${\tau }_{ij}$ and ${e}_{ij}$ are the (i,j)th component of stress tensor and deformation rate respectively, ${\pi }_{c}$ being the critical value of the product $\pi ={e}_{ij}{e}_{ij}$, ${\mu }_{A}$ represents plastic dynamic viscosity and ${p}_{y}$ showing the yield stress. A constant magnetic field is applied perpendicular to the surface. Flow model is then designed by neglecting the effects of induced fields and no external electric field is nearby. The continuity and momentum equations governing equations of the concerned model are as follows^56^:

4 $$\frac{\partial {\widetilde{u}}_{x}}{\partial x}+\frac{\partial {\widetilde{u}}_{y}}{\partial y}=0.$$

5 $$\frac{\partial {\widetilde{u}}_{x}}{\partial t}+{\widetilde{u}}_{x}\frac{\partial {\widetilde{u}}_{x}}{\partial x}+{\widetilde{u}}_{y}\frac{\partial {\widetilde{u}}_{x}}{\partial y}=-\frac{1}{\rho }\frac{\partial `p}{\partial x}+\nu \left(1+\frac{1}{\beta }\right)\left(2\frac{{\partial }^{2}{\widetilde{u}}_{x}}{\partial {x}^{2}}+\frac{{\partial }^{2}{\widetilde{u}}_{x}}{\partial {y}^{2}}+2\frac{{\partial }^{2}{\widetilde{u}}_{y}}{\partial y\partial x}\right)-\frac{\sigma {\mathcal{B}}^{2}}{\rho }{\widetilde{u}}_{x}-\frac{\mu }{\rho k}{\widetilde{u}}_{x}.$$

6 $$\frac{\partial {\widetilde{u}}_{y}}{\partial t}+{\widetilde{u}}_{x}\frac{\partial {\widetilde{u}}_{y}}{\partial x}+{\widetilde{u}}_{y}\frac{\partial {\widetilde{u}}_{y}}{\partial y}=-\frac{1}{\rho }\frac{\partial `p}{\partial y}+\nu \left(1+\frac{1}{\beta }\right)\left(2\frac{{\partial }^{2}{\widetilde{u}}_{y}}{\partial {x}^{2}}+\frac{{\partial }^{2}{\widetilde{u}}_{y}}{\partial {y}^{2}}+2\frac{{\partial }^{2}{\widetilde{u}}_{x}}{\partial y\partial x}\right)-\frac{\mu }{\rho k}{\widetilde{u}}_{y}.$$

where velocity component in x and y direction are ${\widetilde{u}}_{x}$ and ${\widetilde{u}}_{y}$ respectively, $`p$ is pressure, $\mu$ and $\nu$ are dynamic and kinematic viscosities of the fluid respectively, $\beta$=${\mu }_{A}\sqrt{2\pi /{p}_{y}}$ is the Casson fluid parameter, $\mathcal{B}$ and $k$ are imposed magnetic field and permeability constant respectively.

Boundary conditions for the problem are:

7 $$\begin{aligned} {\widetilde{u}}_{x}& =0.\\ {\widetilde{u}}_{y}& ={\widetilde{v}}_{w}=\frac{d\widetilde{h}}{dt}, \;\;at \;\;y=\widetilde{h}(t).\\ \frac{\partial {\widetilde{u}}_{x}}{\partial y}& =0.\\ {\widetilde{u}}_{y}& =0, \;\; at \;\; y=0.\end{aligned}$$

By cross differentiating (5) and (6) gives the following

8 $$\frac{\partial \widetilde{\omega }}{\partial t}+{\widetilde{u}}_{x}\frac{\partial \widetilde{\omega }}{\partial x}+{\widetilde{u}}_{y}\frac{\partial \widetilde{\omega }}{\partial y}=\nu \left(1+\frac{1}{\beta }\right)\left(\frac{{\partial }^{2}\widetilde{\omega }}{\partial {x}^{2}}+\frac{{\partial }^{2}\widetilde{\omega }}{\partial {y}^{2}}\right)-\frac{\sigma {\mathcal{B}}^{2}}{\rho }\frac{\partial {\widetilde{u}}_{x}}{\partial y}-\frac{\mu }{\rho k}\widetilde{\omega }.$$

where

9 $$\widetilde{\omega }=\left(\frac{\partial {\widetilde{u}}_{y}}{\partial x}-\frac{\partial {\widetilde{u}}_{x}}{\partial y}\right).$$

Wang’s^57^ similarity transforms for 2D flow are

10 $$\begin{aligned}{\widetilde{u}}_{x}& =\frac{\gamma x}{2(1-\gamma t)}\mathcal{U} {^{\prime}}(\zeta ).\\ {\widetilde{u}}_{y}& =\frac{-\gamma \mathcalligra{l}}{2(1-\gamma t{)}^{1/2}}\mathcal{U}(\zeta ).\end{aligned}$$

where

$$\zeta =\frac{y}{\mathcalligra{l}\,\,(1-\gamma t{)}^{1/2}}$$

Now by substituting (10) into (8), we have

11 $$\left(1+\frac{1}{\beta }\right)\frac{{d}^{4}\mathcal{U}}{d{\zeta }^{4}}-{S}_{q}\left[\zeta \mathcal{U}+3\frac{{d}^{2}\mathcal{U}}{d{\zeta }^{2}}+\frac{d\mathcal{U}}{d\zeta }\frac{{d}^{2}\mathcal{U}}{d{\zeta }^{2}}-\mathcal{U}\frac{{d}^{3}\mathcal{U}}{d{\zeta }^{3}}\right]-{M}_{g}\frac{{d}^{2}\mathcal{U}}{d{\zeta }^{2}}-{M}_{p}\frac{{d}^{2}\mathcal{U}}{d{\zeta }^{2}}=0.$$

where ${S}_{q}=\frac{\gamma {l}^{2}}{2v}$, ${M}_{g}=\frac{\sigma {\mathcal{B}}^{2}{l}^{2}(1-\gamma t)}{\rho v}$ and ${M}_{p}=\frac{v(1-\gamma t)}{k\gamma }$ are Squeeze, magnetic and permeability parameters respectively. ${S}_{q}<0$ represents that the plates are drawing closer to each other whereas, ${S}_{q}>0$ represents than they are moving farther apart. Using (10), transformed BCs are

12 $$\begin{array}{l}\mathcal{U}(0)=0, \quad \mathcal{U} {^{\prime}} {^{\prime}}(0)=0,\\ \mathcal{U}(1)=1,\quad \mathcal{U} {^{\prime}}(1)=0.\end{array}$$

For $\beta \to \infty$, and ${M}_{g}={M}_{p}$ = 0, problem reduces to the one discussed in^57^.

Skin friction coefficient in this case is^58^

13 $${\mathfrak{C}}_{f}=\nu \left(1+\frac{1}{\beta }\right)\frac{(\frac{\partial {\widetilde{u}}_{x}}{\partial y}{)}_{y=h(t)}}{{\widetilde{v}}_{w}^{2}}.$$

In terms of (10), we have

14 $$\left(1+\frac{1}{\beta }\right)\mathcal{U} {^{\prime}}{^{\prime}}(1)=\frac{{\mathcalligra{l}}^{2}}{{x}^{2}(1-\gamma t)R{e}_{x}{\mathfrak{C}}_{f}}.$$

where

$$R{e}_{x}=\frac{2\mathcalligra{l}{\widetilde{v}}_{w}^{2}}{\nu x(1-\gamma t{)}^{1/2}}$$

In next step, after applying fundamentals of fractional calculus, (11) reduced to the following fractional order differential equation

15 $$\begin{array}{l}\left(1+\frac{1}{\beta }\right)\frac{{d}^{\alpha }\mathcal{U}}{d{\zeta }^{\alpha }}-{S}_{q}\left[\zeta \mathcal{U}+3\frac{{d}^{2}\mathcal{U}}{d{\zeta }^{2}}+\frac{d\mathcal{U}}{d\zeta }\frac{{d}^{2}\mathcal{U}}{d{\zeta }^{2}}-\mathcal{U}\frac{{d}^{3}\mathcal{U}}{d{\zeta }^{3}}\right]-{M}_{g}\frac{{d}^{2}\mathcal{U}}{d{\zeta }^{2}}-{M}_{p}\frac{{d}^{2}\mathcal{U}}{d{\zeta }^{2}}=0.\\ \end{array}$$

where the fractional parameter has range $3<\alpha \le 4$. Equation (15) has the same boundary conditions as in Eq. (12) (Table 1).

**Table 1:** Skin friction coefficient for different values of fractional parameter $\alpha$ when ${M}_{g}$ = 1, ${M}_{p}$ = 2, $\beta$ = 2.4.

Parameter	$\alpha$	$\left(1+\frac{1}{\beta }\right)\mathcal{U} {^{\prime}}{^{\prime}}(1)$
${S}_{q}$ = − 0.3	3.2	$-5.264$
	3.4	$-5.093$
	3.6	$-4.942$
	3.8	$-4.814$
	3.95	$-4.534$
${S}_{q}$ = 0.3	3.2	$-5.545$
	3.4	$-5.363$
	3.6	$-5.195$
	3.8	$-5.047$
	3.95	$-4.948$

## Laplace homotopy perturbation method for fractional differential equations

To illustrate the basic idea of LHPM^59^, consider a general non-linear fractional order differential equation:

16 $${\mathfrak{D}}^{\alpha }[\mathcal{U}(\mathfrak{v})]+\mathfrak{R}[\mathcal{U}(\mathfrak{v})]+\mathcal{N}[\mathcal{U}(\mathfrak{v})]-\mathfrak{g}(\mathfrak{v})=0, \mathfrak{v} \epsilon\Omega , k-1<\alpha \le k.$$

where ${\mathfrak{D}}^{\alpha }[\mathcal{U}(\mathfrak{v})]$ represents the fractional derivative of an unknown function $\mathcal{U}(\mathfrak{v})$, $\mathfrak{g}$($\mathfrak{v}$) is a known function, $\mathfrak{R}$ and $\mathcal{N}$ are linear and non-linear operators respectively. For nth order BVPs, dummy initial conditions are needed to be considered at the start to initialize the solution process.

After considering dummy initial conditions, first step is to apply Laplace transform to both sides of (16), which give the following

17 $$\mathfrak{L}\{{\mathfrak{D}}^{\alpha }[\mathcal{U}(\mathfrak{v})]\}+\mathfrak{L}\{\mathfrak{R}[\mathcal{U}(\mathfrak{v})]+\mathcal{N}[\mathcal{U}(\mathfrak{v})]-\mathfrak{g}(\mathfrak{v})\}=0, k-1<\alpha \le k.$$

By using differential property of Laplace transform, we have

18 $${s}^{\alpha }\mathfrak{L}\{\mathcal{U}(\mathfrak{v})\}-\sum_{n=0}^{k-1}{s}^{\alpha -n-1}{\mathcal{U}}^{(k)}(b)+\mathfrak{L}\{\mathfrak{R}[\mathcal{U}(\mathfrak{v})]+\mathcal{N}[\mathcal{U}(\mathfrak{v})]-\mathfrak{g}(\mathfrak{v})\}=0, k-1<\alpha \le k.$$

or

19 $$\begin{array}{l}\mathfrak{L}\{\mathcal{U}(\mathfrak{v})\}-\left(\frac{1}{{s}^{\alpha }}\right)\sum_{n=0}^{k-1}{s}^{\alpha -n-1}{\mathcal{U}}^{(k)}(b)+\left(\frac{1}{{s}^{\alpha }}\right)\mathfrak{L}\{\mathfrak{R}[\mathcal{U}(\mathfrak{v})]+\mathcal{N}[\mathcal{U}(\mathfrak{v})]-\mathfrak{g}(\mathfrak{v})\}=0,\\ k-1<\alpha \le k.\end{array}$$

In next step of the algorithm, we need to construct a homotopy $\mathcal{V}(\mathfrak{v};\mathfrak{p}):\Omega$ x [0,1] $\to \mathcal{R}$ which satisfies

20 $$\begin{aligned} \mathcal{H} & =(1-\mathfrak{p})(\mathfrak{L}\{\mathcal{V}(\mathfrak{v};\mathfrak{p})\}-{\mathcal{U}}_{0})+\mathfrak{p}(\mathfrak{L}\{\mathcal{V}(\mathfrak{v};\mathfrak{p})\}-\left(\frac{1}{{s}^{\alpha }}\right)\sum_{n=0}^{k-1}{s}^{\alpha -n-1}{\mathcal{U}}^{(k)}(b) \\ & \quad + \left(\frac{1}{{s}^{\alpha }}\right)\mathfrak{L}\{\mathfrak{R}[\mathcal{V}(\mathfrak{v};\mathfrak{p})]+\mathcal{N}[\mathcal{V}(\mathfrak{v};\mathfrak{p}))]-\mathfrak{g}(\mathfrak{v})\}).\end{aligned}$$

where ${\mathcal{U}}_{0}$ is the initial guess which satisfies the given conditions. For $\mathfrak{p}$ = 0 in 20, we have

21 $$\mathfrak{L}\{\mathcal{V}(\mathfrak{v};0)\}-{\mathcal{U}}_{0}=0.$$

and for $\mathfrak{p}$ = 1 in 20 gives the following

22 $$\begin{aligned} & \mathfrak{L}\{\mathcal{V}(\mathfrak{v};1)\}-\left(\frac{1}{{s}^{\alpha }}\right)\sum_{n=0}^{k-1}{s}^{\alpha -n-1}{\mathcal{U}}^{(k)}(b)+\left(\frac{1}{{s}^{\alpha }}\right)\mathfrak{L}\{\mathfrak{R}[\mathcal{V}(\mathfrak{v};1)] \\ & \quad \quad + \mathcal{N}[\mathcal{V}(\mathfrak{v};1))]-\mathfrak{g}(\mathfrak{v})\}=0.\end{aligned}$$

By expanding $\mathcal{V}(\mathfrak{v};\mathfrak{p})$ in terms of Taylor series as:

23 $$\mathcal{V}(\mathfrak{v};\mathfrak{p})=\sum_{n=0}^{\infty }{\mathfrak{p}}^{n}{\mathcal{V}}_{n}.$$

and substituting in Eq. (20), and comparing the coefficients of same powers of $\mathfrak{p}$ leads us to different order problems. The zeroth order problem is:

24 $${\mathfrak{p}}^{0}:\mathfrak{L}\{{\mathcal{V}}_{0}(\mathfrak{v})\}-{\mathcal{U}}_{0}=0.$$

Use of inverse Laplace transform will give the following

25 $${\mathcal{V}}_{0}(\mathfrak{v})={\mathfrak{L}}^{-1}\{{\mathcal{U}}_{0}\}.$$

First order problem is

26 $$\begin{aligned} & {\mathfrak{p}}^{1}:\mathfrak{L}\{{\mathcal{V}}_{1}(\mathfrak{v})\}+{\mathcal{U}}_{0}-\left(\frac{1}{{s}^{\alpha }}\right)\sum_{n=0}^{k-1}{s}^{\alpha -n-1}{\mathcal{U}}^{(k)}(b)+\left(\frac{1}{{s}^{\alpha }}\right)\mathfrak{L}\{\mathfrak{R}[{\mathcal{V}}_{0}(\mathfrak{v})] \\ & \quad \quad + \mathcal{N}[{\mathcal{V}}_{0}(\mathfrak{v}))]-\mathfrak{g}(\mathfrak{v})\}=0.\end{aligned}$$

Application of inverse Laplace transform leads to

27 $$\begin{aligned} {\mathcal{V}}_{1}(\mathfrak{v})&={\mathfrak{L}}^{-1}\left\{-{\mathcal{U}}_{0}+\left(\frac{1}{{s}^{\alpha }}\right)\sum_{n=0}^{k-1}{s}^{\alpha -n-1}{\mathcal{U}}^{(k)}(b)\right\} \\ & \quad- {\mathfrak{L}}^{-1}\left\{\left(\frac{1}{{s}^{\alpha }}\right)\mathfrak{L}\{\mathfrak{R}[{\mathcal{V}}_{0}(\mathfrak{v})]+\mathcal{N}[{\mathcal{V}}_{0}(\mathfrak{v})]-\mathfrak{g}(\mathfrak{v})\}\right\}.\end{aligned}$$

Similarly second order problem is

28 $${\mathfrak{p}}^{2}:\mathfrak{L}\{{\mathcal{V}}_{2}(\mathfrak{v})\}+\left(\frac{1}{{s}^{\alpha }}\right)\mathfrak{L}\{\mathfrak{R}[{\mathcal{V}}_{1}(\mathfrak{v})]+\mathcal{N}[{\mathcal{V}}_{1}(\mathfrak{v})]\}=0.$$

Use of inverse Laplace transform give the following

29 $${\mathcal{V}}_{2}(\mathfrak{v})=-{\mathfrak{L}}^{-1}\left\{\left(\frac{1}{{s}^{\alpha }}\right)\mathfrak{L}\{\mathfrak{R}[{\mathcal{V}}_{1}(\mathfrak{v})]+\mathcal{N}[{\mathcal{V}}_{1}(\mathfrak{v})]\}\right\}.$$

continuing this way, we can get higher order problems and their solutions. The approximate solution of the general fractional order, non-linear differential equation is

30 $$\widetilde{\mathcal{V}}=\underset{\mathfrak{p}\to 1}{\mathrm{lim}}\mathcal{V}={\mathcal{V}}_{0}+{\mathcal{V}}_{1}+{\mathcal{V}}_{2}+{\mathcal{V}}_{3}+\cdots$$

**Figure 1:**
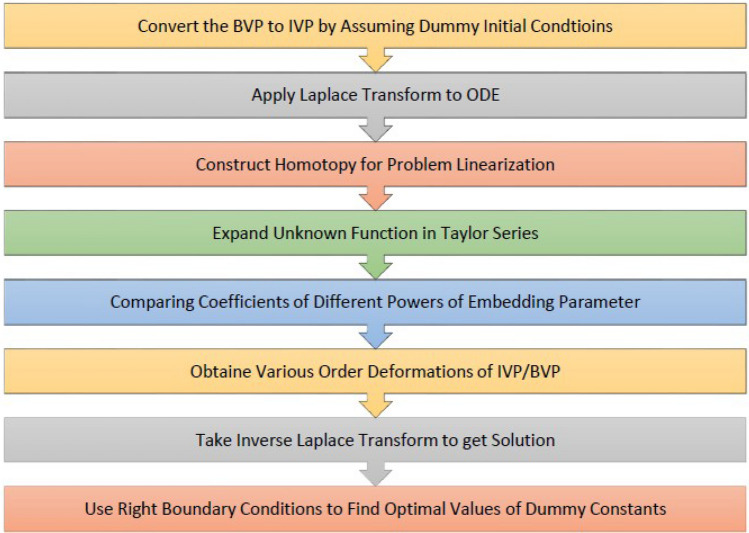
Block diagram of LPHM procedure.

Since dummy constants are introduced at the start of solution process in case of BVPs. Right boundary conditions will use for finding optimal values of dummies. The block diagram of LHPM procedure is given above in Fig. 1. Residual error can be found by substituting approximate solution in concerned differential equation as

31 $$Res={\mathfrak{D}}^{\alpha }[\widetilde{\mathcal{V}}]+\mathfrak{R}[\widetilde{\mathcal{V}}]+\mathcal{N}[\widetilde{\mathcal{V}}]-\mathfrak{g}(\mathfrak{v}).$$

## Application of LHPM to fractional squeezing of Casson fluid

Firstly, apply Laplace transform on both sides of (15) as

32 $$\begin{array}{l}\mathfrak{L}\left\{\left(1+\frac{1}{\beta }\right)\frac{{d}^{\alpha }\mathcal{U}}{d{\zeta }^{\alpha }}\right\}-\mathfrak{L}\left\{{S}_{q}\left[\zeta \mathcal{U}+3\frac{{d}^{2}\mathcal{U}}{d{\zeta }^{2}}+\frac{d\mathcal{U}}{d\zeta }\frac{{d}^{2}\mathcal{U}}{d{\zeta }^{2}}-\mathcal{U}\frac{{d}^{3}\mathcal{U}}{d{\zeta }^{3}}\right]-{M}_{g}\frac{{d}^{2}\mathcal{U}}{d{\zeta }^{2}}-{M}_{p}\frac{{d}^{2}\mathcal{U}}{d{\zeta }^{2}}\right\}=0,\\ \quad 3<\alpha \le 4.\end{array}$$

Utilization of differential property of Laplace transform gives the following form

33 $$\begin{array}{l}\left(1+\frac{1}{\beta }\right)\left({s}^{\alpha }\mathfrak{L}\{\mathcal{U}(\zeta )\}-\sum_{n=0}^{k-1}{s}^{\alpha -n-1}{\mathcal{U}}^{(k)}(0)\right)-\mathfrak{L}\left\{{S}_{q}\left[\zeta \mathcal{U}+3\frac{{d}^{2}\mathcal{U}}{d{\zeta }^{2}}+\frac{d\mathcal{U}}{d\zeta }\frac{{d}^{2}\mathcal{U}}{d{\zeta }^{2}}-\mathcal{U}\frac{{d}^{3}\mathcal{U}}{d{\zeta }^{3}}\right]\right\}\\ \quad -\mathfrak{L}\left\{{M}_{g}\frac{{d}^{2}\mathcal{U}}{d{\zeta }^{2}}+{M}_{p}\frac{{d}^{2}\mathcal{U}}{d{\zeta }^{2}}\right\}=0.\end{array}$$

In next step, construct a homotopy using (15) as

34 $$\begin{array}{l}\mathcal{H}=(1-\mathfrak{p})(\mathfrak{L}\{\mathcal{U}(\zeta )\}-{\mathcal{U}}_{0})+\mathfrak{p}(\mathfrak{L}\{\mathcal{U}(\zeta )\}-\left(\frac{1}{{s}^{\alpha }}\right)\sum_{n=0}^{k-1}{s}^{\alpha -n-1}{\mathcal{U}}^{(k)}(0)-\mathfrak{L}\{\left(\frac{1+\beta }{\beta }\right)\left(\frac{1}{{s}^{\alpha }}\right)\\ \left({S}_{q}\left[\zeta \mathcal{U}+3\frac{{d}^{2}\mathcal{U}}{d{\zeta }^{2}}+\frac{d\mathcal{U}}{d\zeta }\frac{{d}^{2}\mathcal{U}}{d{\zeta }^{2}}-\mathcal{U}\frac{{d}^{3}\mathcal{U}}{d{\zeta }^{3}}\right]-{M}_{g}\frac{{d}^{2}\mathcal{U}}{d{\zeta }^{2}}-{M}_{p}\frac{{d}^{2}\mathcal{U}}{d{\zeta }^{2}}\right)\}).\end{array}$$

Further, by expanding $\mathcal{U}(\zeta )$ as a Taylor series, and comparing the coefficients of like powers of $\mathfrak{p}$ will give different order problems as follows:

Zeroth-order deformation

35 $$\mathfrak{L}\{{\mathcal{U}}_{0}(\zeta )\}-{\mathcal{U}}_{0}=0,$$

$${\mathcal{U}}_{0}(0)=0,$$

$$\mathcal{U}{{^{\prime}}}_{0}\left(0\right)={a}_{1},$$

$$\mathcal{U}{{^{\prime}}{^{\prime}}}_{0}(0)=0,$$

$$\mathcal{U}{{^{\prime}}{^{\prime}}{^{\prime}}}_{0}\left(0\right)={a}_{2}.$$

First-order deformation

36 $$\begin{array}{l}\mathfrak{L}\{{\mathcal{U}}_{1}(\zeta )\}+{\mathcal{U}}_{0}-\left(\frac{1}{{s}^{\alpha }}\right)\sum_{n=0}^{k-1}{s}^{\alpha -n-1}{\mathcal{U}}_{1}^{(k)}(0)-\mathfrak{L}\{\left(\frac{1+\beta }{\beta }\right)\left(\frac{1}{{s}^{\alpha }}\right)({S}_{q}[\zeta {\mathcal{U}}_{0}+3\frac{{d}^{2}{\mathcal{U}}_{0}}{d{\zeta }^{2}}\\ \quad +\frac{d{\mathcal{U}}_{0}}{d\zeta }\frac{{d}^{2}{\mathcal{U}}_{0}}{d{\zeta }^{2}}-{\mathcal{U}}_{0}\frac{{d}^{3}{\mathcal{U}}_{0}}{d{\zeta }^{3}}]-{M}_{g}\frac{{d}^{2}{\mathcal{U}}_{0}}{d{\zeta }^{2}}-{M}_{p}\frac{{d}^{2}{\mathcal{U}}_{0}}{d{\zeta }^{2}})\}.\end{array}$$

$${\mathcal{U}}_{1}(0)=0,$$

$$\mathcal{U}{{^{\prime}}}_{1}(0)=0,$$

$$\mathcal{U}{{^{\prime}}{^{\prime}}}_{1}(0)=0,$$

$$\mathcal{U}{{^{\prime}}{^{\prime}}{^{\prime}}}_{1}(0)=0.$$

mth-order deformation

37 $$\begin{array}{l}\mathfrak{L}\{{\mathcal{U}}_{m}(\zeta )\}-\mathfrak{L}\{\left(\frac{1+\beta }{\beta }\right)\left(\frac{1}{{s}^{\alpha }}\right)({S}_{q}\left[\zeta {\mathcal{U}}_{m-1}+3\frac{{d}^{2}{\mathcal{U}}_{m-1}}{d{\zeta }^{2}}+\frac{d{\mathcal{U}}_{m-1}}{d\zeta }\frac{{d}^{2}{\mathcal{U}}_{m-1}}{d{\zeta }^{2}}-{\mathcal{U}}_{m-1}\frac{{d}^{3}{\mathcal{U}}_{m-1}}{d{\zeta }^{3}}\right]\\\quad -{M}_{g}\frac{{d}^{2}{\mathcal{U}}_{m-1}}{d{\zeta }^{2}}-{M}_{p}\frac{{d}^{2}{\mathcal{U}}_{m-1}}{d{\zeta }^{2}})\}.\end{array}$$

$${\mathcal{U}}_{m}(0)=0,$$

$$\mathcal{U}{{^{\prime}}}_{m}(0)=0,$$

$$\mathcal{U}{{^{\prime}}{^{\prime}}}_{m}(0)=0,$$

$$\mathcal{U}{{^{\prime}}{^{\prime}}{^{\prime}}}_{m}(0)=0.$$

Application of inverse Laplace transform on the above-mentioned problems will lead to ${\mathcal{U}}_{0},{\mathcal{U}}_{1},{\mathcal{U}}_{2}$ $\cdots$

Thus, approximate solution is

$$\widetilde{\mathcal{U}}={\mathcal{U}}_{0}+{\mathcal{U}}_{1}+{\mathcal{U}}_{2}+{\mathcal{U}}_{3}+\dots$$

It is to note that, optimal values of unknown dummy conditions ${a}_{1} \; \; \mathrm{and} \; \; {a}_{2}$ be determined by using right boundary conditions from (12). After this, putting obtained solution back to (15) for obtaining residual function:

38 $$Res=\left(1+\frac{1}{\beta }\right)\frac{{d}^{\alpha }\widetilde{\mathcal{U}}}{d{\zeta }^{\alpha }}-{S}_{q}\left[\zeta \widetilde{\mathcal{U}}+3\frac{{d}^{2}\widetilde{\mathcal{U}}}{d{\zeta }^{2}}+\frac{\widetilde{\mathcal{U}}}{d\zeta }\frac{{d}^{2}\widetilde{\mathcal{U}}}{d{\zeta }^{2}}-\widetilde{\mathcal{U}}\frac{{d}^{3}\widetilde{\mathcal{U}}}{d{\zeta }^{3}}\right]-{M}_{g}\frac{{d}^{2}\widetilde{\mathcal{U}}}{d{\zeta }^{2}}-{M}_{p}\frac{{d}^{2}\widetilde{\mathcal{U}}}{d{\zeta }^{2}}.$$

## Results and discussion

In this article, unsteady squeezing flow of Casson fluid passing through a porous medium is considered in fractional space. An acquired highly nonlinear boundary value problem is solved and analyzed with respect to fractional parameter *α*,$3<\alpha \le 4$, which was introduced because of fractional environment. Main focus of the paper is to investigate the effect of fractional parameter on fluid velocity and skin friction. Moreover, comparative analysis of the behaviors of different fluid parameters ($\beta ,{M}_{g},{M}_{p},{S}_{q}$) on the velocity profiles in fractional and integral environment is the second aspect of this investigation. Modeled problem is solved using Laplace transform with homotopy perturbation for above-mentioned parameters, and obtained results are compared with HPM. Table 2 and 3 show LHPM solutions and errors for different values of $\alpha$ when squeeze number ${S}_{q}$ is negative and positive respectively. Tables 4, 5, 6, 7 provide the comparison of LHPM results with HPM. Analysis of these tables reveals that LHPM results are consistent and are better than HPM. Moreover, skin friction for fixed values of fluid parameters in fractional scenario is also determined in this study, and numerical results are presented in Table 1. It is observed that skin friction decreases with an increase in $\alpha$, for positive and negative squeeze number.

**Table 2:** LHPM solutions and errors for different $\alpha$ when ${S}_{q}$ is negative i.e. ${S}_{q}=-1$, and ${M}_{g}=0.5$, ${M}_{p}=1.5$, $\beta =0.05$.

$\zeta$	$\alpha =3.3$		$\alpha =3.5$		$\alpha =3.7$		$\alpha =3.9$	
	LHPM	Residual error	LHPM	Residual error	LHPM	Residual error	LHPM	Residual error
$0.0$	$0.0$	$0.0$	$0.0$	$0.0$	$0.0$	$0.0$	$0.0$	$0.0$
$0.2$	$0.29610$	$6.1\times 1{0}^{-10}$	$0.29612$	$1.6\times 1{0}^{-10}$	$0.29612$	$3.6\times 1{0}^{-11}$	$0.29611$	$6.9\times 1{0}^{-12}$
$0.4$	$0.56812$	$1.2\times 1{0}^{-8}$	$0.56816$	$5.6\times 1{0}^{-9}$	$0.56817$	$2.0\times 1{0}^{-9}$	$0.56816$	$6.0\times 1{0}^{-10}$
$0.6$	$0.79206$	$3.6\times 1{0}^{-8}$	$0.79211$	$3.4\times 1{0}^{-8}$	$0.79213$	$1.8\times 1{0}^{-8}$	$0.79213$	$7.1\times 1{0}^{-9}$
$0.8$	$0.94400$	$3.7\times 1{0}^{-8}$	$0.94403$	$8.5\times 1{0}^{-8}$	$0.94404$	$6.7\times 1{0}^{-8}$	$0.94404$	$3.5\times 1{0}^{-8}$
$1.0$	$1.0$	$5.5\times 1{0}^{-7}$	$1.0$	$6.4\times 1{0}^{-8}$	$1.0$	$1.5\times 1{0}^{-7}$	$1.0$	$1.0\times 1{0}^{-7}$

**Table 3:** LHPM solutions and errors for different $\alpha$ when ${S}_{q}$ is positive i.e. ${S}_{q}=1$, and ${M}_{g}=0.5$, ${M}_{p}=1.5$, $\beta =0.05$.

$\zeta$	$\alpha =3.3$		$\alpha =3.5$		$\alpha =3.7$		$\alpha =3.9$	
	LHPM	Residual error	LHPM	Residual error	LHPM	Residual error	LHPM	Residual error
$0.0$	$0.0$	$0.0$	$0.0$	$0.0$	$0.0$	$0.0$	$0.0$	$0.0$
$0.2$	$0.29416$	$8.9\times 1{0}^{-10}$	$0.29441$	$1.1\times 1{0}^{-10}$	$0.29467$	$1.8\times 1{0}^{-13}$	$0.29492$	$2.6\times 1{0}^{-12}$
$0.4$	$0.56531$	$3.8\times 1{0}^{-8}$	$0.56565$	$7.4\times 1{0}^{-9}$	$0.56601$	$5.0\times 1{0}^{-10}$	$0.56636$	$1.8\times 1{0}^{-10}$
$0.6$	$0.78979$	$4.2\times 1{0}^{-7}$	$0.79004$	$1.1\times 1{0}^{-7}$	$0.79032$	$1.6\times 1{0}^{-8}$	$0.79059$	$3.1\times 1{0}^{-10}$
$0.8$	$0.94312$	$2.6\times 1{0}^{-6}$	$0.94321$	$8.0\times 1{0}^{-7}$	$0.94331$	$1.8\times 1{0}^{-7}$	$0.94341$	$2.3\times 1{0}^{-8}$
$1.0$	$1.0$	$1.1\times 1{0}^{-5}$	$1.0$	$4.0\times 1{0}^{-6}$	$1.0$	$1.2\times 1{0}^{-6}$	$1.0$	$2.6\times 1{0}^{-7}$

**Table 4:** Comparison of LHPM and HPM results in case of negative ${S}_{q}$ when $\alpha =4.0$, ${M}_{g}={M}_{p}=0.5,\beta =0.05$.

Parameter	$\eta$	Solution		Residual error	
		HPM^56^	LHPM	HPM^56^	LHPM
${S}_{q}=-0.2$	$0.1$	$0.14944$	$0.14944$	$9.93\times 1{0}^{-11}$	$4.35\times 1{0}^{-16}$
	$0.3$	$0.43635$	$0.43635$	$5.16\times 1{0}^{-10}$	$9.73\times 1{0}^{-13}$
	$0.5$	$0.68732$	$0.68732$	$5.33\times 1{0}^{-10}$	$3.56\times 1{0}^{-11}$
	$0.7$	$0.87838$	$0.87838$	$1.53\times 1{0}^{-9}$	$3.82\times 1{0}^{-10}$
	$0.9$	$0.98547$	$0.98547$	$1.17\times 1{0}^{-9}$	$2.25\times 1{0}^{-9}$
${S}_{q}=-0.6$	$0.1$	$0.14955$	$0.14955$	$1.24\times 1{0}^{-8}$	$3.06\times 1{0}^{-15}$
	$0.3$	$0.43664$	$0.43664$	$3.79\times 1{0}^{-8}$	$5.95\times 1{0}^{-12}$
	$0.5$	$0.68765$	$0.68765$	$7.21\times 1{0}^{-8}$	$1.78\times 1{0}^{-10}$
	$0.7$	$0.87859$	$0.87859$	$1.42\times 1{0}^{-7}$	$1.47\times 1{0}^{-9}$
	$0.9$	$0.98556$	$0.98551$	$3.01\times 1{0}^{-7}$	$6.26\times 1{0}^{-9}$

**Table 5:** Comparison of LHPM and HPM results for different $\beta$ when $\alpha =4.0$, ${M}_{g}={M}_{p}=0.5,{S}_{q}=-0.2$.

Parameter	$\eta$	Solution		Residual error	
		HPM^56^	LHPM	HPM^56^	LHPM
$\beta =0.01$	$0.1$	$0.14948$	$0.14948$	$8.92\times 1{0}^{-13}$	$8.67\times 1{0}^{-19}$
	$0.3$	$0.43646$	$0.43646$	$4.63\times 1{0}^{-12}$	$1.82\times 1{0}^{-15}$
	$0.5$	$0.68746$	$0.68746$	$4.79\times 1{0}^{-12}$	$6.67\times 1{0}^{-14}$
	$0.7$	$0.87847$	$0.87847$	$1.37\times 1{0}^{-11}$	$7.17\times 1{0}^{-13}$
	$0.9$	$0.98549$	$0.98549$	$1.05\times 1{0}^{-11}$	$4.21\times 1{0}^{-12}$
$\beta =0.2$	$0.1$	$0.14929$	$0.14929$	$4.25\times 1{0}^{-9}$	$6.47\times 1{0}^{-14}$
	$0.3$	$0.43597$	$0.43597$	$2.21\times 1{0}^{-8}$	$1.45\times 1{0}^{-10}$
	$0.5$	$0.68690$	$0.68690$	$2.28\times 1{0}^{-8}$	$5.30\times 1{0}^{-9}$
	$0.7$	$0.87810$	$0.87810$	$6.57\times 1{0}^{-8}$	$5.70\times 1{0}^{-8}$
	$0.9$	$0.98542$	$0.98542$	$5.05\times 1{0}^{-8}$	$8.38\times 1{0}^{-7}$

**Table 6:** Comparison of LHPM and HPM results for different ${M}_{g}$ when $\alpha =4.0$, $\beta =0.05$, ${M}_{p}=0.5,{S}_{q}=-0.2$.

Parameter	$\eta$	Solution		Residual error	
		HPM^56^	LHPM	HPM^56^	LHPM
${M}_{g}=0.1$	$0.1$	$0.14937$	$0.14948$	$3.30\times 1{0}^{-8}$	$3.12\times 1{0}^{-18}$
	$0.3$	$0.43617$	$0.43646$	$8.69\times 1{0}^{-8}$	$1.87\times 1{0}^{-14}$
	$0.5$	$0.68713$	$0.68746$	$1.08\times 1{0}^{-7}$	$1.22\times 1{0}^{-12}$
	$0.7$	$0.87826$	$0.87847$	$8.46\times 1{0}^{-8}$	$1.86\times 1{0}^{-11}$
	$0.9$	$0.98545$	$0.98549$	$9.68\times 1{0}^{-9}$	$1.36\times 1{0}^{-10}$
${M}_{g}=1.0$	$0.1$	$0.14926$	$0.14938$	$2.69\times 1{0}^{-7}$	$2.34\times 1{0}^{-15}$
	$0.3$	$0.43591$	$0.43620$	$6.78\times 1{0}^{-7}$	$5.16\times 1{0}^{-12}$
	$0.5$	$0.68683$	$0.68716$	$7.49\times 1{0}^{-7}$	$1.86\times 1{0}^{-10}$
	$0.7$	$0.87807$	$0.87827$	$3.99\times 1{0}^{-7}$	$1.98\times 1{0}^{-9}$
	$0.9$	$0.98542$	$0.98546$	$2.89\times 1{0}^{-7}$	$1.16\times 1{0}^{-8}$

**Table 7:** Comparison of LHPM and HPM results for different ${M}_{p}$ when $\alpha =4.0$, $\beta =0.05$, ${M}_{g}=0.5,{S}_{q}=-0.2$.

Parameter	$\eta$	Solution		Residual error	
		HPM^56^	LHPM	HPM^56^	LHPM
${M}_{p}=1.0$	$0.1$	$0.14938$	$0.14938$	$2.87\times 1{0}^{-9}$	$2.34\times 1{0}^{-15}$
	$0.3$	$0.43620$	$0.43620$	$2.56\times 1{0}^{-9}$	$5.16\times 1{0}^{-12}$
	$0.5$	$0.68716$	$0.68716$	$6.02\times 1{0}^{-9}$	$1.86\times 1{0}^{-10}$
	$0.7$	$0.87827$	$0.87827$	$6.00\times 1{0}^{-9}$	$1.98\times 1{0}^{-9}$
	$0.9$	$0.98546$	$0.98546$	$1.14\times 1{0}^{-8}$	$1.16\times 1{0}^{-8}$
${M}_{p}=1.7$	$0.1$	$0.14930$	$0.14930$	$4.79\times 1{0}^{-8}$	$1.02\times 1{0}^{-14}$
	$0.3$	$0.43599$	$0.43599$	$8.64\times 1{0}^{-8}$	$2.24\times 1{0}^{-11}$
	$0.5$	$0.68692$	$0.68692$	$1.60\times 1{0}^{-10}$	$8.05\times 1{0}^{-10}$
	$0.7$	$0.87812$	$0.87812$	$7.43\times 1{0}^{-8}$	$8.54\times 1{0}^{-9}$
	$0.9$	$0.98543$	$0.98543$	$3.80\times 1{0}^{-9}$	$4.99\times 1{0}^{-8}$

**Figure 2:**
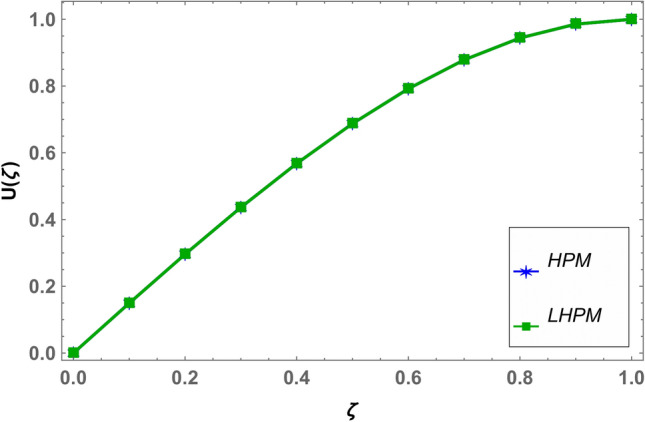
Comparison of LHPM and HPM^56^ solutions when $\alpha =4$, ${S}_{q}=-0.4$, $\beta =1.0$, ${M}_{g}=0.5$ and ${M}_{p}=0.5$.

**Figure 3:**
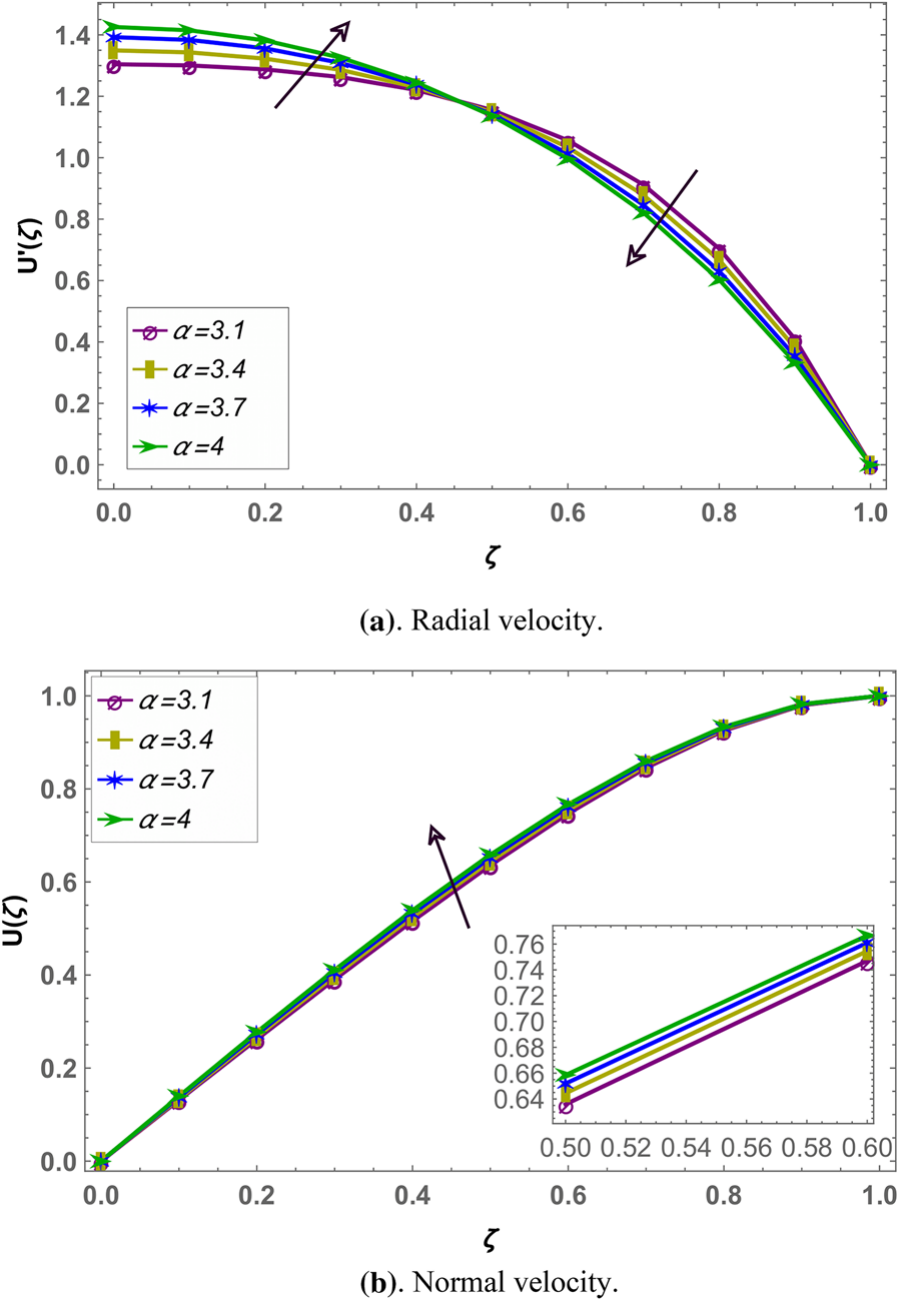
Effect of fractional parameter $\alpha$ on the velocity profile when ${S}_{q}$ is negative i.e. ${S}_{q}=-1$, and ${M}_{g}=3,{M}_{p}=3.5$ and $\beta =4$ are fixed.

**Figure 4:**
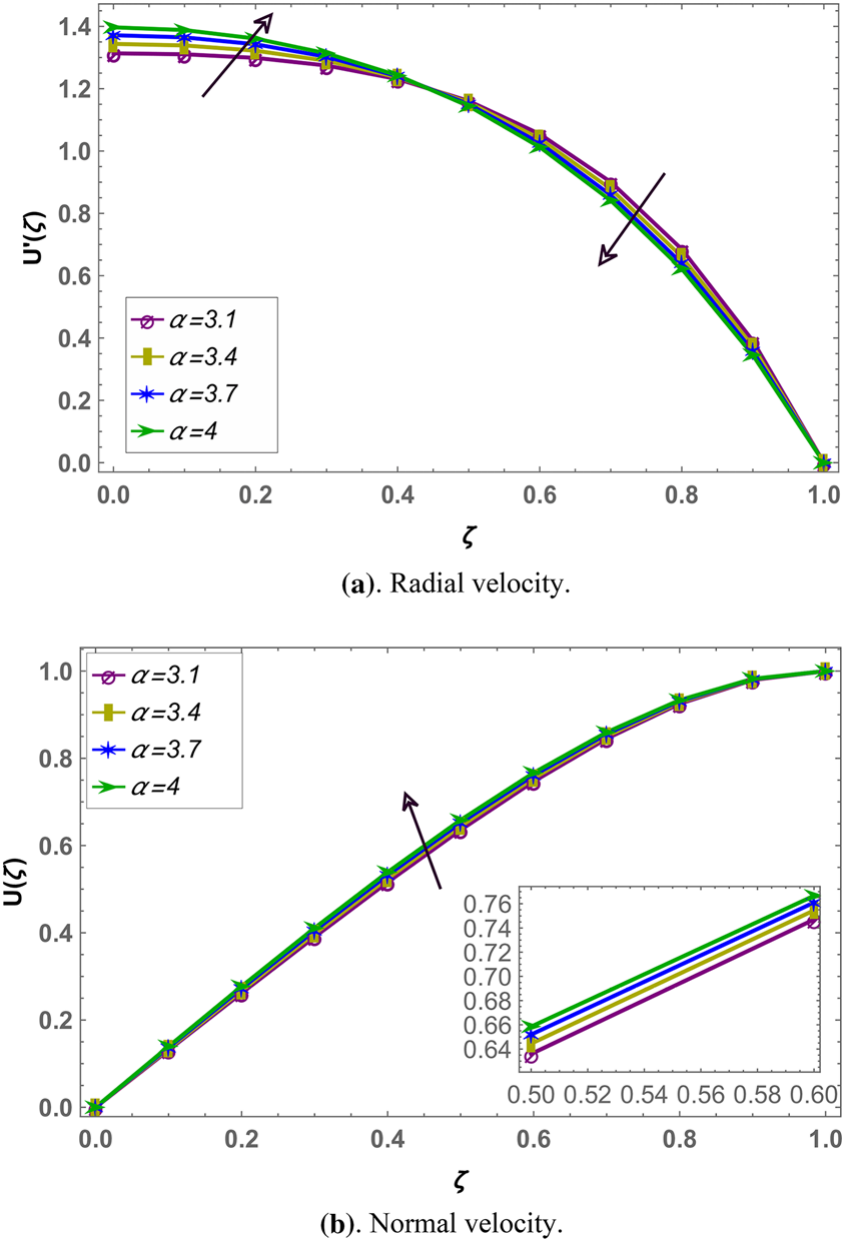
Effect of fractional parameter $\alpha$ on the velocity profile when ${S}_{q}$ is positive i.e. ${S}_{q}=5$, and ${M}_{g}=3,{M}_{p}=3.5$ and $\beta =0.5$ fixed.

**Figure 5:**
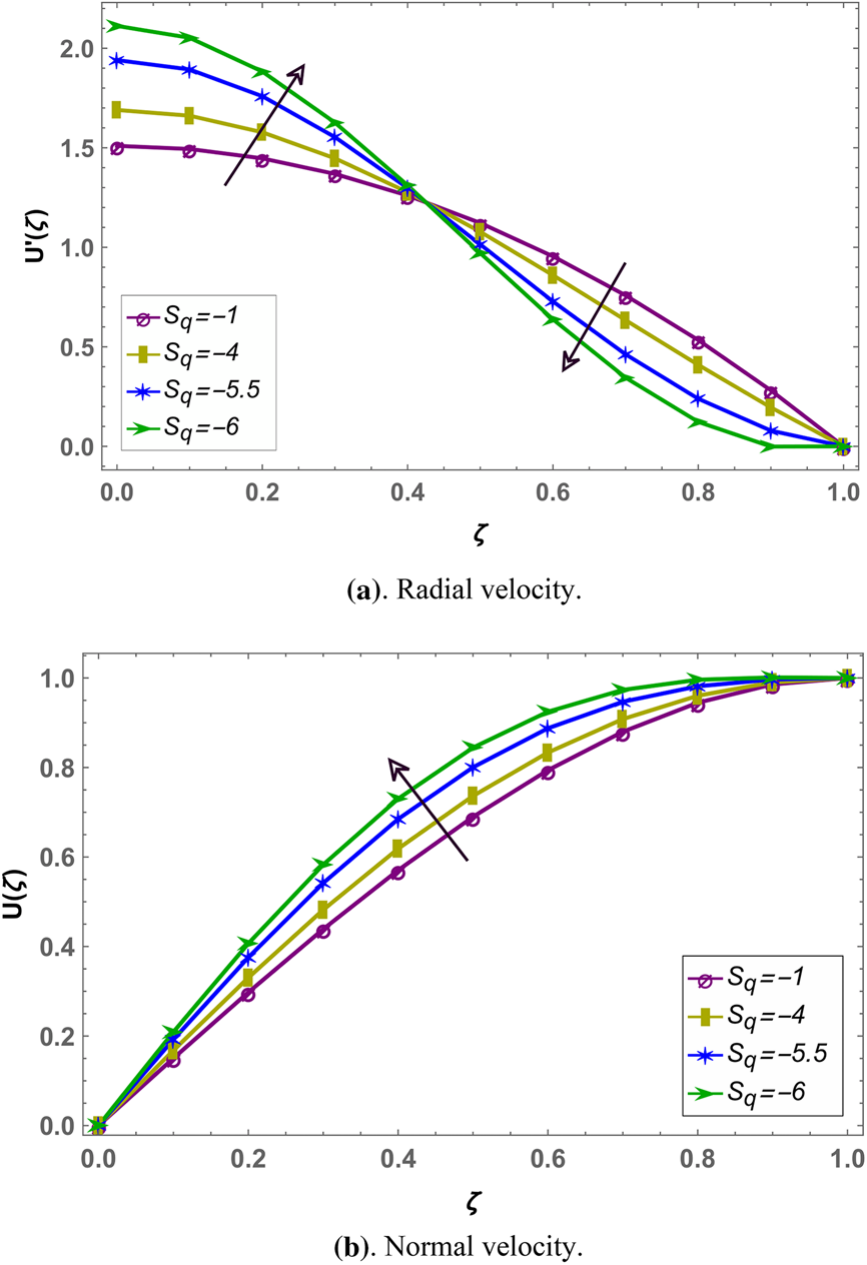
Effect of negative squeeze number ${S}_{q}$ on the velocity profile with $\alpha =3.6$, ${M}_{g}=0.9$, ${M}_{p}=0.8$ and $\beta =0.5$.

**Figure 6:**
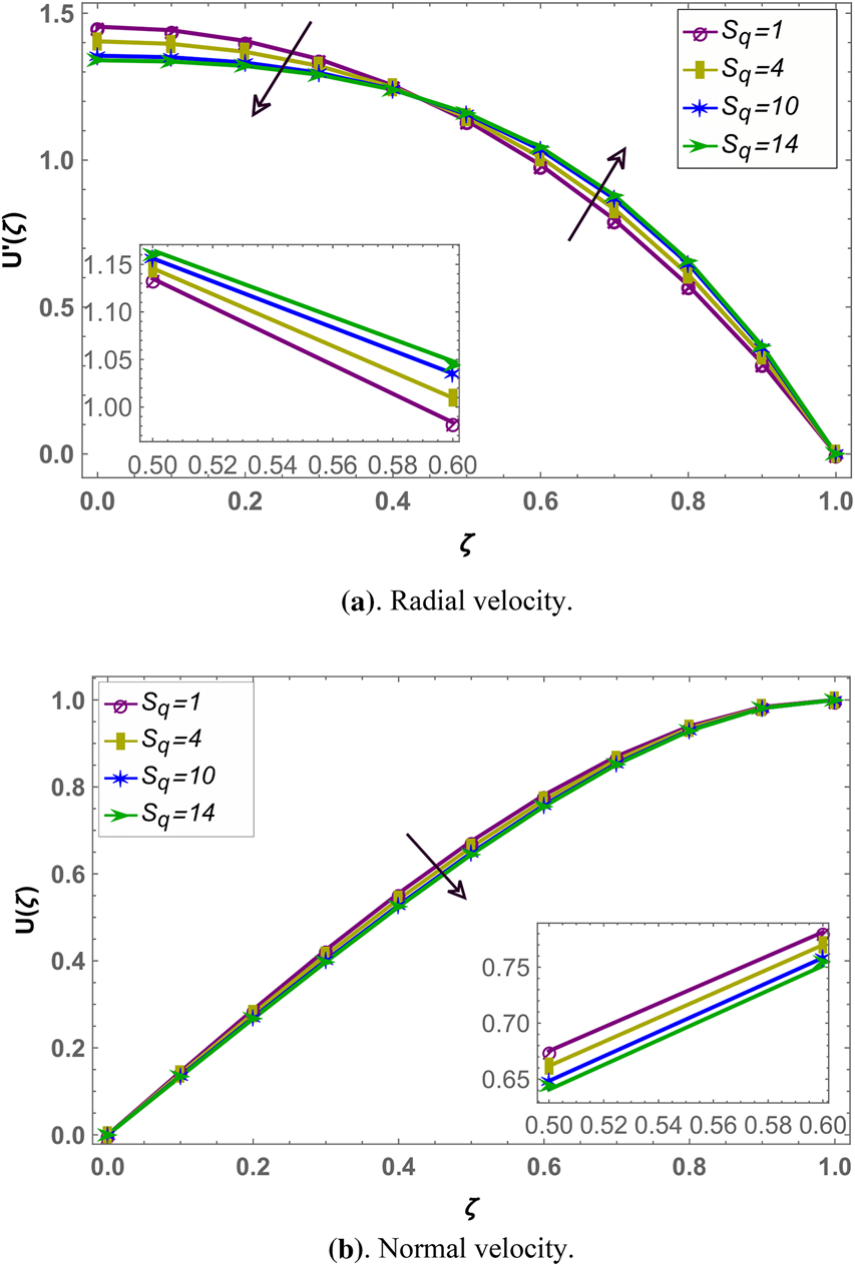
Effect of positive squeeze number ${S}_{q}$ on the velocity profile with $\alpha =3.6$, ${M}_{g}=0.9$, ${M}_{p}=0.8$ and $\beta =0.5.$

Beside numerical analysis of the results, validity of the obtained solution is confirmed by comparing it with existing solution which can be seen in Fig. 2. Also, effects of different fluid parameters on the velocity profile are similar graphically in fractional environment for the cases of positive squeeze numbers (plates are moving farther apart from each other) and negative squeeze numbers (plates are moving towards each other). It has also been observed that Casson parameter shows opposite behavior in fractional and integral domain when squeeze number is negative. Rest of the parameters are showing similar behavior in fractional and integral scenarios^56^. Figures 3 and 4 demonstrate the effect of fractional parameter on the velocity profile in both cases when the distance between plates is increasing or decreasing. It is observed that fractional parameter showed similar effect in both the cases. It is seen that normal velocity increases with an increase of fractional parameter whereas the radial velocity increases when $\zeta \in (0.0.5)$ and decreases onward. Effect of negative and positive squeeze number ${S}_{q}$ on the velocity profile in fractional environment is displayed in Figs. 5 and 6 respectively. . It is seen that in case of negative ${S}_{q}$, normal velocity increases with an increase in negative ${S}_{q}$ while radial velocity increases when $\zeta \in (\mathrm{0,0.5})$ and decreases onward. Opposite behavior is recorded when positive ${S}_{q}$ (see Fig. 6).

**Figure 7:**
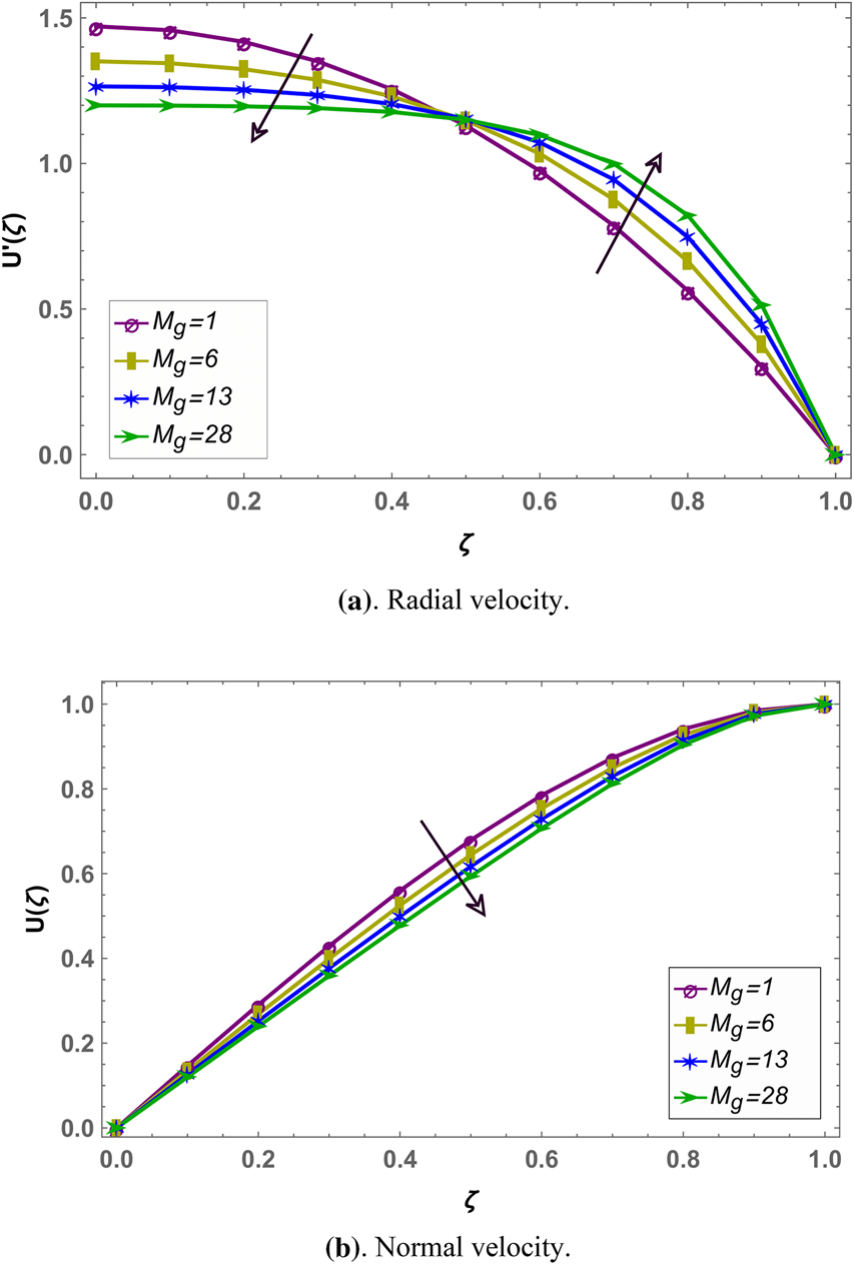
Effect of MHD parameter ${M}_{g}$ on the velocity when ${S}_{q}$ is negative i.e. ${S}_{q}=-0.3$, $\alpha =3.6$, ${M}_{p}=0.6$ and $\beta =5$.

**Figure 8:**
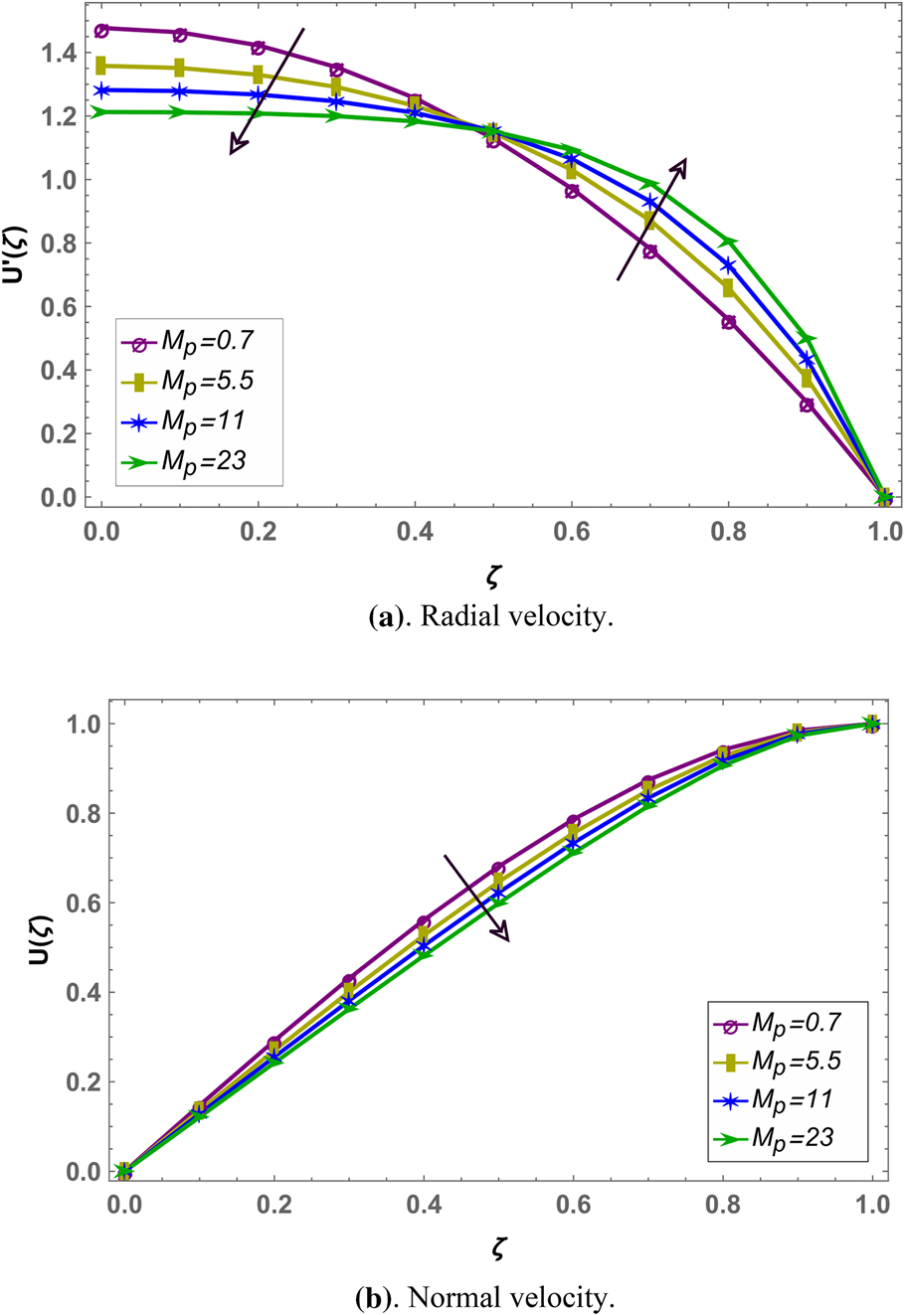
Effect of permeability parameter ${M}_{p}$ on the velocity when ${S}_{q}$ is negative i.e. ${S}_{q}=-0.3$, $\alpha =3.6$, ${M}_{g}=0.7$ and $\beta =5$.

**Figure 9:**
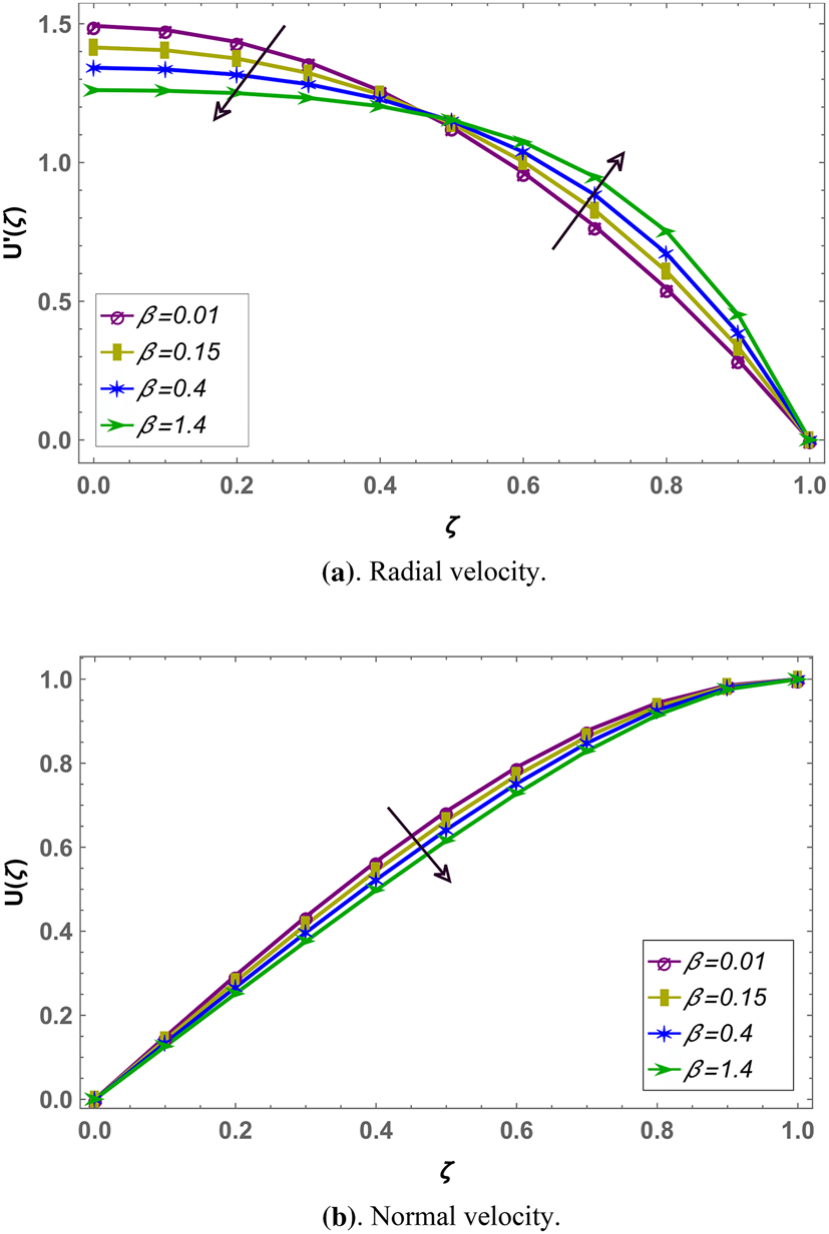
Effect of Casson parameter $\beta$ on the velocity when ${S}_{q}$ is negative i.e. ${S}_{q}=-0.3$, $\alpha =3.6$, ${M}_{g}=10$ and ${M}_{p}=10$.

**Figure 10:**
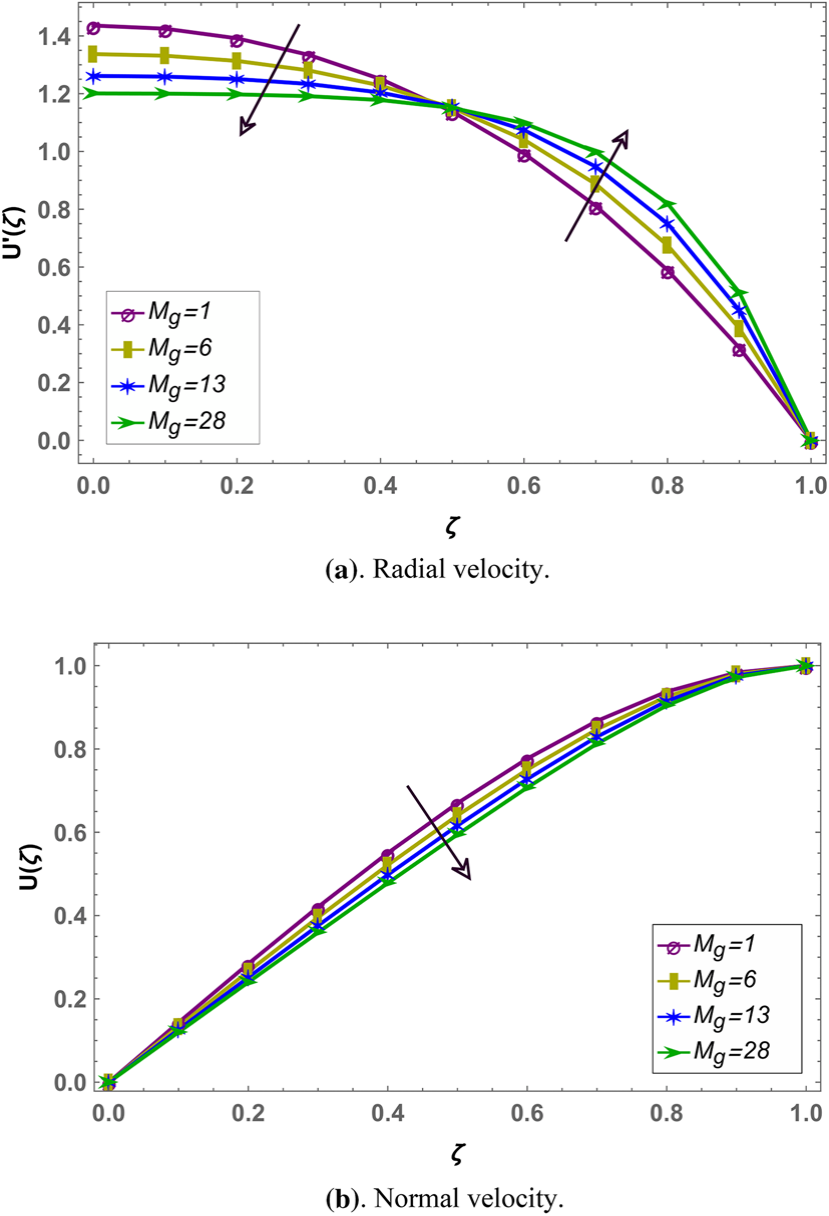
Effect of MHD parameter ${M}_{g}$ on the velocity when ${S}_{q}$ is positive i.e. ${S}_{q}=0.3$, $\alpha =3.6$, ${M}_{p}=0.6$ and $\beta =5$.

**Figure 11:**
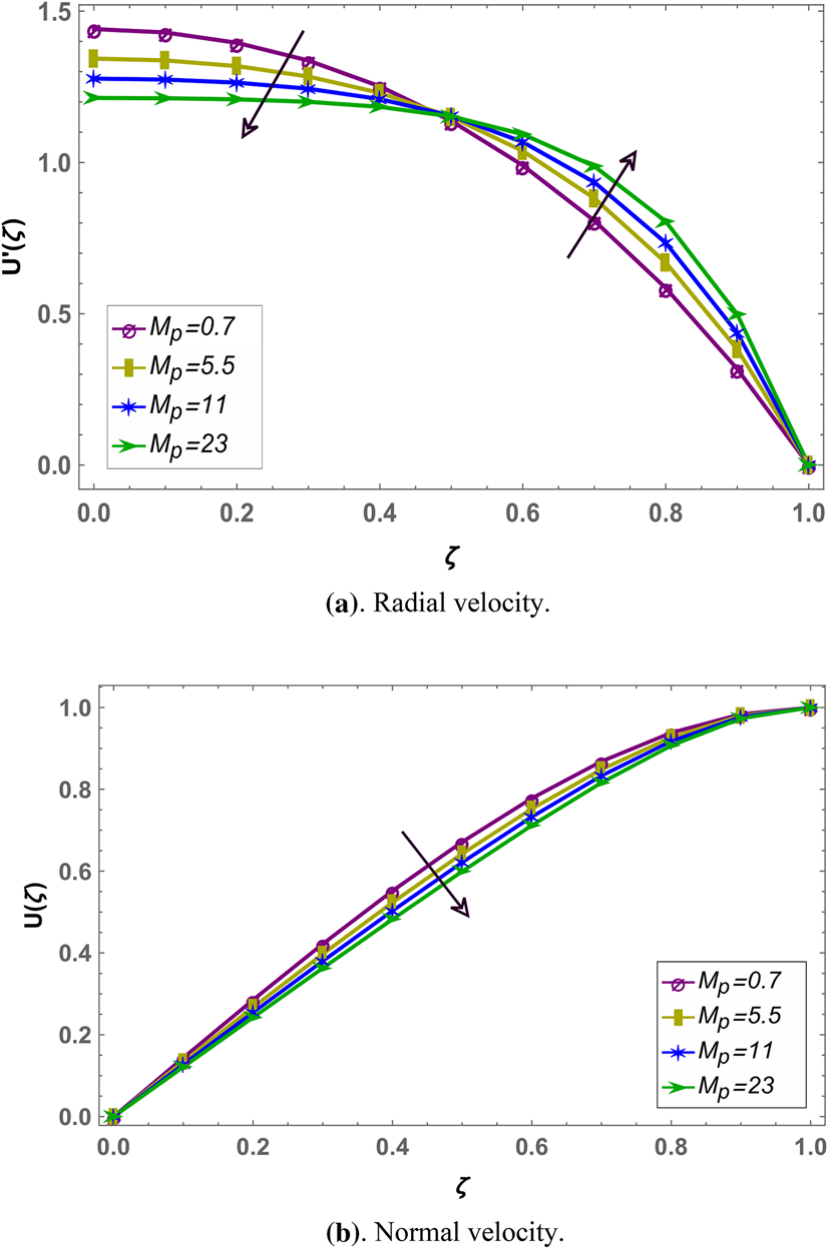
Effect of permeability parameter ${M}_{p}$ on the velocity when ${S}_{q}$ is positive i.e. ${S}_{q}=0.3$, $\alpha =3.6$, ${M}_{g}=0.7$ and $\beta =5$.

**Figure 12:**
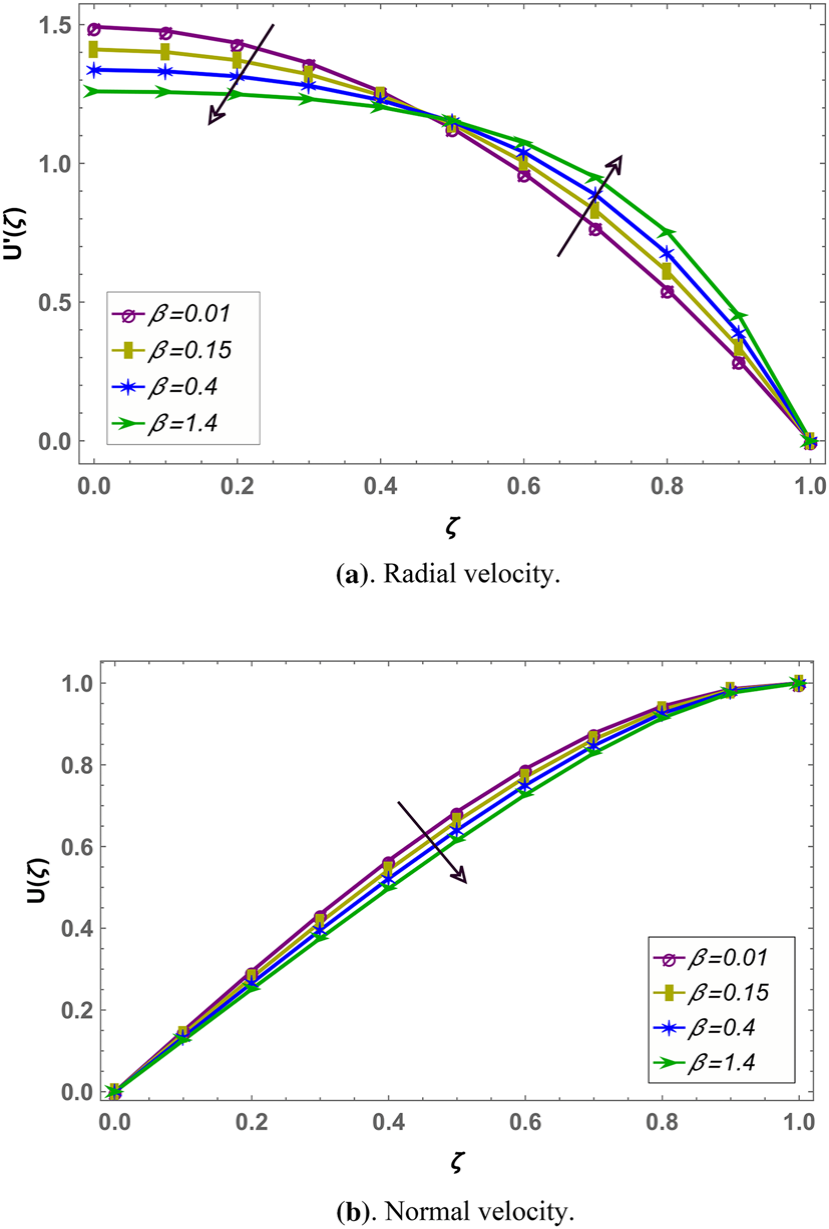
Effect of Casson parameters $\beta$ on the velocity profile when ${S}_{q}$ is positive i.e. ${S}_{q}=0.3$, $\alpha =3.6$, ${M}_{g}=10$ and ${M}_{p}=10$.

Effect of magnetohydrodynamic parameter ${M}_{g}$ on normal and radial velocity in case of positive and negative squeeze number is given in Figs. 7 and 10 respectively in fractional environment. Under increasing magnetic force, Lorentz like drag force increases which decreases velocity. It is observed that increasing magnetic effect shows similar behavior in both squeezing or receding case. It is observed that normal velocity decreases with an increase in ${M}_{g}$ while radial velocity decreases when $\zeta \in (\mathrm{0,0.5})$ and increases onward. Similar effects are reported in rest of the fluid parameters (i.e. ${M}_{p},\beta$) in fractional environment See (Figs. 8, 9, 10, 11, 12). It is observed that ${M}_{p}$ and $\beta$ are behaving similarly (like ${M}_{g}$) on the normal and radial components of velocity whether ${S}_{q}$ is negative or positive.

## Conclusion

This article is based on fractional analysis of unsteady squeezing flow of MHD Casson fluid passing through a porous channel. Obtained highly non-linear fractional order boundary value problem is solved using Laplace transform along with homotopy perturbation. For validity of the obtained results, residual errors are calculated in fractional and integral environments. Moreover, obtained results shows good agreement with available solutions from the literature. It is also noted that LHPM provides better accuracy than existing results in literature, therefore, it can be extended to other non-Newtonian fluid models such as Oldryod 6, Carreau and Sutterby fluid etc. Moreover, Casson fluid model in fractional calculus can be studied at various boundary conditions of fluid solid interface. Analysis of the concerned model leads to the following key findings:

- Skin friction decreases with an increase in fraction parameter.
- Fractional parameters behave similarly on the velocity profile in case of negative and positive squeeze number.
- Effects of various fluid parameters in fractional space are analyzed graphically and it is observed that, Casson parameter is the only one who behave differently in fractional and integral environment, i.e. Casson parameter showed similar effect in fractional environment for negative and positive squeeze number but has shown opposite effects in integral environment.

The Homotopy Perturbation method could be applied to a variety of physical and technical challenges in the future^60–71^. Some recent developments exploring the significance of the considered research domain are reported by^72–81^.

## Data Availability

All data generated or analyzed during this study are included in this published article.
